# Impact of pneumothorax on mortality, morbidity, and hospital resource utilization in COVID-19 patients: a propensity matched analysis of nationwide inpatient sample database

**DOI:** 10.1186/s12890-024-03161-z

**Published:** 2024-07-31

**Authors:** Adeel Nasrullah, Mohammed A. Quazi, Shiza Virk, Sitara Niranjan, Muhammad Ali Butt, Muhammad Hassan Shakir, Amir Humza Sohail, Muhammad Ibraiz Bilal, Taimur Muzammil, Briana DiSilvio, Abu Baker Sheikh, Tariq Cheema

**Affiliations:** 1 Division of Pulmonary and Critical Care, Allegheny Health Network, Pittsburgh, PA 15212 USA; 2 Department of Psychiatry and Behavioral Sciences, University of New Mexico Health Sciences Center, Albuquerque, NM 87131 USA; 3 Department of Internal Medicine, Allegheny Health Network, Pittsburgh, PA 15212 USA; 4 Department of Internal Medicine, University of New Mexico Health Sciences Center, Albuquerque, NM 87131 USA; 5 Department of Internal Medicine, The Wright Center for Graduate Medical Education, Scranton, PA 18505 USA

**Keywords:** COVID-19, SARS-CoV-2, Pneumothorax, Complications, Mortality, Prevalence, National inpatient sample

## Abstract

**Background:**

Spontaneous pneumothorax (PTX) is more prevalent among COVID-19 patients than other critically ill patients, but studies on this are limited. This study compared clinical characteristics and in-hospital outcomes among COVID-19 patients with concomitant PTX to provide insight into how PTX affects health care utilization and complications, which informs clinical decisions and healthcare resource allocation.

**Methods:**

The 2020 Nationwide Inpatient Sample was used analyze patient demographics and outcomes, including age, race, sex, insurance status, median income, length of hospital stay, mortality rate, hospitalization costs, comorbidities, mechanical ventilation, and vasopressor support. Propensity score matching was employed for additional analysis.

**Results:**

Among 1,572,815 COVID-19 patients, 1.41% had PTX. These patients incurred significantly higher hospitalization costs ($435,508 vs. $96,668, *p* < 0.001) and longer stays (23.6 days vs. 8.6 days, *p* < 0.001). In-hospital mortality was substantially elevated for PTX patients (65.8% vs. 14.4%, *p* < 0.001), with an adjusted odds ratio of 14.3 (95% CI 12.7–16.2). Additionally, these patients were more likely to require vasopressors (16.6% vs. 3.3%), mechanical circulatory support (3.5% vs. 0.3%), hemodialysis (16.6% vs. 5.6%), invasive mechanical ventilation (76.9% vs. 15.1%), non-invasive mechanical ventilation (19.1% vs. 5.8%), tracheostomy (13.3% vs. 1.1%), and chest tube placement (59.8% vs. 0.8%).

**Conclusions:**

Our findings highlight the severe impact of PTX on COVID-19 patients, characterized by higher mortality, more complications, and increased resource utilization. Also, being Hispanic, male, or obese increased the risk of developing concomitant PTX with COVID-19.

**Supplementary Information:**

The online version contains supplementary material available at 10.1186/s12890-024-03161-z.

## Background

Coronavirus disease is caused by a highly infectious pathogen infection and can present with minimal, non-specific symptoms [1, 2]. ( Severe COVID-19 is characterized by acute respiratory distress syndrome (ARDS)-like hyperinflammation and endothelial dysfunction, that can lead to respiratory failure in the acute phase or as a post-Covid syndrome [3, 4].

The Coronavirus Disease 2019 (COVID-19) has increased morbidity and mortality rates up to 40% in critically ill patients, and to combat this, we must understand the disease’s complications, especially those that are potentially life-threatening [5, 6]. One such complication is pneumothorax (PTX), a condition observed in both spontaneously breathing patients and those on mechanical ventilation [7]. PTX is a potentially crippling complication of COVID-19, with an average reported incidence ranging from 1 to 10%, depending on clinical severity [8, 9]. There are few studies that address the impact of PTX on morbidity, mortality, and resource utilization among COVID-19 patients, highlighting a significant gap in our understanding [10, 11].

Before the COVID-19 pandemic, PTX was a common and potentially fatal complication in mechanically ventilated patients (MVPs) with acute respiratory distress syndrome (ARDS), with an overall 30-day mortality rate of 40% [12]. Mortality was largely attributed to air leaks and acute lung injury. The traditionally high ventilatory volumes and high airway pressure in the diseased lungs caused volutrauma and barotrauma, respectively [13]. The overall incidence of PTX decreased from 55 to 17% after the implementation of protective lung strategies [14]. Other factors related to the occurrence of PTX include history of chronic pulmonary disease, smoking, and ARDS [15].

Research indicates a higher occurrence of PTX in COVID-19 patients independent of their mechanical ventilation status. COVID-19 patients on mechanical ventilation have higher rates of barotrauma and volutrauma progressing to PTX than patients with conventional ARDS or other viral pneumonias [16–18]. Spontaneous PTX is more prevalent among COVID-19 patients than other critically ill individuals, with incidence rates ranging from 0.3 to 23.8% in the COVID-19 positive population [8, 9, 19, 20]. There are also reports of patients with non-severe COVID-19 developing spontaneous PTX [21–23].

We assessed the influence of PTX on COVID-19 patient mortality rates, morbidity, and healthcare resource utilization. A better understanding of the effect of PTX on COVID-19 patient prognosis will guide treatment of this high-risk cohort. The strategic implementation of preventative strategies may ultimately reduce hospital expenditures and improve outcomes in the COVID-19 positive population.

## Methods

This was a retrospective study conducted using Nationwide Inpatient Sample (NIS) data from 2020. International classification of diseases 10th—clinical modification (ICD-10-CM) codes were used to retrieve patient samples with comorbid conditions, and ICD-10 procedure codes were used to identify inpatient procedures (Supplemental Table 1). All COVID-19 positive patients ≥ 18 years of age admitted to the hospital were included in this study. Variables were divided into patient-related, hospital-related, and indicators of illness severity. We collected patient age, race, sex, insurance status (Medicare, Medicaid, private insurance, self-payment, no charge), median income based on patient’s zip code, and disposition. We also collected hospital location, teaching status, bed size, and region. Lastly, we collected data indicating illness severity, including length of stay (LOS), mortality, hospitalization cost, comorbidities, and treatment interventions. The primary outcome of our study was in-hospital mortality, and the secondary outcomes included intubation and mechanical ventilation, hemodialysis use, mechanical circulatory support use, vasopressor use, non-invasive ventilation, chest tube placement, tracheostomy, cardiovascular accident, cardiogenic shock, venous thromboembolism, acute liver failure, sudden cardiac arrest, LOS, the financial burden on healthcare, and resource utilization.

### Statistical analysis

For descriptive statistics in Table 1, continuous variables are summarized as mean ± standard deviation (SD), and categorical data are expressed as numbers and percentages. We used the Rao-Scott Chi-square test for categorical variables (e.g., sex and risk factors) to assess the independence in Table 1, and a multivariate linear regression model for continuous variables (LOS and total hospitalization cost). Moreover, we developed a multivariable logistic regression model for each categorical variable in Table 2 to report adjusted odds ratios for the two cohorts. We used a threshold of *p* ≤ 0.20 to retain predictor variables in the multivariable logistic regression models and *p* ≤ 0.05 in the multivariate linear regression model. The multivariable logistic regression models and the multivariate linear regression model used hospital-related, Elixhauser comorbidities, and patient-related variables as predictors. Since, the control group (COVID-19 positive but PTX-negative) had a significantly higher sample than the test group (COVID-19 positive and PTX-positive), we conducted a secondary, propensity score matched analysis (PSM) to confirm results obtained on the raw samples. Baseline demographics (age, race, sex, income status, insurance status) were matched using a 1:1 nearest neighbor propensity score with 0.05 caliper width. On matched cohorts, the same analysis was repeated with the same regression models. The results from the PSM samples are reported in Tables 3 and 4. All analyses were performed using R, SAS, and Python programming language.

**Table 1:** Demographics and characteristics for patients with COVID-19 without pneumothorax and COVID-19 with pneumothorax

Characteristics	COVID-19 without Pneumothorax		COVID-19 with Pneumothorax		*P* value
	*N*	%	*N*	%	
*N* = 1,572,815	1,550,585	98.59	22,230	1.41	--
Gender (%)	*N*	%	*N*	%	< 0.001
Female	750,250	48.38	7600	34.19	
Male	800,335	51.62	14,630	65.81	
Mean age (years)	Mean	SD	Mean	SD	< 0.01
Female	63.08	18.90	65.91	14.01	
Male	63.51	16.34	63.47	13.86	
Age groups (%)	*N*	%	*N*	%	< 0.001
18–29	77,310	4.99	430	1.93	
30–49	260,605	16.81	2855	12.84	
50–69	572,960	36.95	10,380	46.69	
>=70	639,710	41.26	8565	38.53	
Race (%)	*N*	%	*N*	%	< 0.001
Asian or Pacific	50,640	3.27	775	3.49	
Black	296,955	19.15	2855	12.84	
Hispanic	330,615	21.32	7060	31.76	
Native American	14,510	0.94	320	1.44	
Other	65,985	4.26	1390	6.25	
White	791,880	51.07	9830	44.22	
Median household income (%)	*N*	%	*N*	%	0.004
<= 49,999	528,455	34.08	8100	36.44	
50k-64,999	420,260	27.10	5985	26.92	
65k-85,999	344,215	22.20	4745	21.35	
>=86k	257,655	16.62	3400	15.29	
Insurance status (%)	*N*	%	*N*	%	0.113
Medicaid	222,135	14.33	3110	13.99	
Medicare	789,720	50.93	10,925	49.15	
No charge	4180	0.27	65	0.29	
Other	67,510	4.35	1040	4.68	
Private Insurance	408,925	26.37	6225	28.00	
Self-pay	58,115	3.75	865	3.89	
Hospital region (%)	*N*	%	*N*	%	< 0.001
East North Central	243,815	15.72	2795	12.57	
East South Central	106,065	6.84	1490	6.70	
Middle Atlantic	231,875	14.95	3565	16.04	
Mountain	98,080	6.33	1685	7.58	
New England	59,970	3.87	525	2.36	
Pacific	175,495	11.32	2750	12.37	
South Atlantic	318,425	20.54	4310	19.39	
West North Central	95,990	6.19	1085	4.88	
West South Central	220,870	14.24	4025	18.11	
Hospital size (%)	*N*	%	*N*	%	< 0.001
Large	723,110	46.63	11,195	50.36	
Medium	449,575	28.99	6785	30.52	
Small	377,900	24.37	4250	19.12	
Hospital teaching status (%)	*N*	%	*N*	%	< 0.001
Rural	148,995	9.61	1270	5.71	
Urban nonteaching	293,305	18.92	4000	17.99	
Urban teaching	1,108,285	71.48	16,960	76.29	
Comorbidities (%)	*N*	%	*N*	%	
CAD	277,475	17.89	3340	15.02	< 0.001
MI	65,350	4.21	695	3.12	< 0.001
HTN	1,006,520	64.91	13,925	62.64	0.001
Diabetes	621,075	40.05	9255	41.63	0.032
Cancer	64,745	4.18	1100	4.95	0.010
Obesity	409,610	26.42	6355	28.59	0.001
Drug Abuse	29,180	1.88	405	1.82	0.769
Smoking	397,205	25.62	4365	19.64	< 0.001
Alcohol	35,255	2.27	445	2.00	< 0.001
Chronic Pulmonary Disease	335,340	21.63	4780	21.50	0.841
Peripheral Vascular Disease	62,805	4.05	915	4.11	0.825
CKD	196,450	12.67	2365	10.64	< 0.001
Hypothyroidism	205,760	13.27	2330	10.48	< 0.001
Autoimmune	48,025	3.09	755	3.39	0.253
Depression	170,000	10.96	1680	7.55	< 0.001
Dementia	188,460	12.15	1295	5.82	< 0.001
AIDS	7735.00	0.49	95.00	0.42	0.501

CAD: coronary artery disease, MI: myocardial infarction, HTN: hypertension, CKD: Chronic kidney disease, AIDS: acquired immune deficiency syndrome

**Table 2:** In-hospital outcomes for patients with COVID-19 without pneumothorax and COVID-19 with pneumothorax

Characteristics	COVID-19 without Pneumothorax		COVID-19 with Pneumothorax		*P* value
	N	%	N	%	
*N* = 1,572,815	1,550,585	98.59	22,230	1.41	--
Disposition (%)	*N*	%	*N*	%	< 0.001
Against medical advice	17,490	1.13	75	0.34	
Died in Hospital	196,015	12.64	14,625	65.79	
Discharged alive unknown destination	900	0.06	5	0.02	
Home health care	203,090	13.10	1190	5.35	
Routine	798,955	51.53	1685	7.58	
Transfer other	288,215	18.59	3650	16.42	
Transfer to short-term hospital	45,920	2.96	1000	4.50	
Complications (%)	*N*	%	*N*	%	
In hospital mortality (*N* = 210,640)	196,015	12.64	14,625	65.78	< 0.001
	Adjusted odds ratio* = 15.89 (95% CI 14.86–17.00)				
Acute Liver Failure	14,585	0.94	1640	7.37	< 0.001
	Adjusted odds ratio* = 7.21 (95% CI 6.39–8.13)				
Sudden Cardiac Arrest	38,130	2.45	3620	16.28	< 0.001
	Adjusted odds ratio* = 7.02 (95% CI 6.45–7.64)				
Vasopressor use	37,505	2.41	3685	16.57	< 0.001
	Adjusted odds ratio* = 7.15 (95% CI 6.57–7.79)				
Mechanical Circulatory Support	3450	0.22	780	3.50	< 0.001
	Adjusted odds ratio* = 13.99 (95% CI 11.62–16.82)				
AKI	436,660	28.16	13,480	60.63	< 0.001
	Adjusted odds ratio* = 4.35 (95% CI 4.09–4.64)				
VTE	69,790	4.50	3300	14.84	< 0.001
	Adjusted odds ratio* = 3.50 (95% CI 3.21–3.81)				
Cardiogenic shock	8985	0.57	630	2.83	< 0.001
	Adjusted odds ratio* = 4.23 (95% CI 3.52–5.10)				
Hemodialysis	76,340	4.92	3690	16.59	< 0.001
	Adjusted odds ratio* = 3.97 (95% CI 3.64–4.33)				
Invasive Mechanical Ventilation	180,150	11.61	17,105	76.94	< 0.001
	Adjusted odds ratio* = 24.49 (95% CI 22.78–26.32)				
Non-Invasive Mechanical Ventilation	85,925	5.54	4235	19.05	< 0.001
	Adjusted odds ratio* = 3.78 (95% CI 3.49–4.08)				
CVA	25,020	1.61	1060	4.76	< 0.001
	Adjusted odds ratio* = 2.89 (95% CI 2.50–3.33)				
Tracheostomy	14,475	0.93	2960	13.31	< 0.001
	Adjusted odds ratio* = 14.06 (95% CI 12.76–15.49)				
Chest Tube Placement	9700	0.62	13,285	59.76	< 0.001
	Adjusted odds ratio* = 244.93 (95% CI 226.31–265.07)				
Quality parameters					
Mean total hospitalization charge ($)	$86,822.22		$435,507.54		< 0.001
	Adjusted total charge* = $336782.46 higher for Pneumothorax+				
Mean length of stay (days)	7.79		23.59		< 0.001
	Adjusted length of stay* = 15.34 days higher for Pneumothorax+				

AKI: Acute kidney injury, VTE: Venous Thromboembolism, CVA: Cerebrovascular Accident

**Table 3:** Propensity-matched analysis of demographics and patient characteristics

Characteristics	COVID-19 without Pneumothorax		COVID-19 with Pneumothorax		*P* value
	*N*	%	*N*	%	
*N* = 44,460	22,230	50.00	22,230	50.00	--
Gender (%)	*N*	%	*N*	%	0.575
Female	7475	33.62	7600	34.19	
Male	14,755	66.37	14,630	65.81	
Mean age (years)	Mean	SD	Mean	SD	
Female	65.74	14.95	65.91	14.01	
Male	63.91	14.26	63.47	13.86	
Age groups (years)	*N*	%	*N*	%	0.172
18–29	515	2.32	430	1.93	
30–49	2825	12.70	2855	12.84	
50–69	9950	44.75	10,380	46.69	
>=70	8940	40.21	8565	38.53	
Race (%)	*N*	%	*N*	%	0.046
Asian or Pacific	730	3.28	775	3.49	
Black	2800	12.60	2855	12.84	
Hispanic	7175	32.28	7060	31.76	
Native American	175	0.79	320	1.44	
Other	1250	5.62	1390	6.25	
White	10,100	45.43	9830	44.22	
Median household income (%)	*N*	%	*N*	%	0.626
<= 49,999	8225	37.00	8100	36.44	
50k-64,999	6080	27.35	5985	26.92	
65k-85,999	4740	21.32	4745	21.35	
>=86k	3185	14.33	3400	15.29	
Insurance status (%)	*N*	%	*N*	%	0.992
Medicaid	3110	13.99	3110	13.99	
Medicare	11,065	49.78	10,925	49.15	
No charge	60	0.27	65	0.29	
Other	1040	4.68	1040	4.68	
Private Insurance	6095	27.42	6225	28.00	
Self-pay	860	3.87	865	3.89	
Hospital Region (%)	*N*	%	*N*	%	< 0.001
East North Central	3570	16.06	2795	12.57	
East South Central	445	2.00	1490	6.70	
Middle Atlantic	7425	33.40	3565	16.04	
Mountain	665	2.99	1685	7.58	
New England	3600	16.19	525	2.36	
Pacific	1250	5.62	2750	12.37	
South Atlantic	2815	12.66	4310	19.39	
West North Central	880	3.96	1085	4.88	
West South Central	1580	7.11	4025	18.11	
Hospital size (%)	*N*	%	*N*	%	< 0.001
Large	9845	44.29	11,195	50.36	
Medium	6355	28.59	6785	30.52	
Small	6030	27.13	4250	19.12	
Hospital teaching status (%)	*N*	%	*N*	%	< 0.001
Rural	1685	7.58	1270	5.71	
Urban nonteaching	3030	13.63	4000	17.99	
Urban teaching	17,515	78.79	16,960	76.29	
Comorbidities (%)	*N*	%	*N*	%	
CAD	4130	15.57	3340	15.02	< 0.001
MI	955	4.29	695	3.12	0.003
HTN	14,120	63.51	13,925	62.64	0.391
Diabetes	9270	41.70	9255	41.63	0.948
Cancer	935	4.20	1100	4.95	0.094
Obesity	6145	27.64	6355	28.59	0.321
Drug Abuse	250	1.12	405	1.82	0.006
Smoking	6005	27.01	4365	19.64	< 0.001
Alcohol	390	1.75	445	2.00	0.390
Chronic Pulmonary Disease	4480	20.15	4780	21.50	0.117
Peripheral Vascular Disease	770	3.46	915	4.11	0.107
CKD	2705	12.16	2365	10.64	0.023
Hypothyroidism	2210	9.94	2330	10.48	0.400
Autoimmune	575	2.58	755	3.39	0.025
Depression	1555	6.99	1680	7.55	0.307
Dementia	1195	5.37	1295	5.82	0.356
AIDS	120	0.53	95.00	0.42	0.444

CAD: coronary artery disease, MI: myocardial infarction, HTN: hypertension, CKD: Chronic kidney disease, AIDS: acquired immune deficiency syndrome

**Table 4:** Propensity-matched analysis of in-hospital outcomes

Characteristics	COVID-19 without Pneumothorax		COVID-19 with Pneumothorax		*P* value
	*N*	%	*N*	%	
*N* = 44,460	22,230	50.00	22,230	50.00	--
Disposition (%)	*N*	%	*N*	%	< 0.001
Against medical advice	195	0.88	75	0.34	
Died in Hospital	3200	14.39	14,625	65.79	
Discharged alive unknown destination	20	0.09	5	0.02	
Home health care	3205	14.42	1190	5.35	
Routine	11,150	50.16	1685	7.58	
Transfer other	3610	16.24	3650	16.42	
Transfer to short-term hospital	850	3.82	1000	4.50	
Complications	*N*	%	*N*	%	
Acute Liver Failure	260	1.17	1640	7.37	< 0.001
	Adjusted odds ratio* = 6.70 (95% CI 4.98–9.02)				
Sudden Cardiac Arrest	585	2.63	3620	16.28	< 0.001
	Adjusted odds ratio* = 7.40 (95% CI 5.98–9.15)				
In hospital mortality (*N* = 17,825)	3200	14.39	14,625	65.78	< 0.001
	Adjusted odds ratio* = 14.33 (95% CI 12.69–16.18)				
Vasopressor use	725	3.26	3685	16.57	< 0.001
	Adjusted odds ratio* = 7.48 (95% CI 6.15–9.10)				
Mechanical Circulatory Support	65	0.29	780	3.50	< 0.001
	Adjusted odds ratio* = 11.50 (95% CI 6.41–20.63)				
AKI	6675	30.02	13,480	60.63	< 0.001
	Adjusted odds ratio* = 4.06 (95% CI 3.68–4.49)				
VTE	1165	5.24	3300	14.84	< 0.001
	Adjusted odds ratio* = 3.21 (95% CI 2.72–3.80)				
Cardiogenic shock	125	0.56	630	2.83	< 0.001
	Adjusted odds ratio* = 5.11 (95% CI 3.31–7.89)				
Hemodialysis	1250	5.62	3690	16.59	< 0.001
	Adjusted odds ratio* = 3.73 (95% CI 3.16–4.40)				
Invasive Mechanical Ventilation	3360	15.11	17,105	76.94	< 0.001
	Adjusted odds ratio* = 23.12 (95% CI 20.39–26.21)				
Non-Invasive Mechanical Ventilation	1300	5.84	4235	19.05	< 0.001
	Adjusted odds ratio* = 3.84 (95% CI 3.28–4.50)				
CVA	365	1.64	1060	4.76	< 0.001
	Adjusted odds ratio* = 3.02 (95% CI 2.30–3.97)				
Tracheostomy	245	1.10	2960	13.31	< 0.001
	Adjusted odds ratio* = 14.15 (95% CI 10.52–19.04)				
Chest Tube Placement	170	0.76	13,285	59.76	< 0.001
	Adjusted odds ratio* = 231.29 (95% CI 163.09–328.02)				
Quality parameters					
Mean total hospitalization charge ($)	$96,667.97		$435,507.54		< 0.001
	Adjusted total charge* = $320977.60 higher for Pneumothorax+				
Mean length of stay (days)	8.56		23.59		< 0.001
	Adjusted length of stay* = 14.94 days higher for Pneumothorax+				

AKI: Acute kidney injury, VTE: Venous Thromboembolism, CVA: Cerebrovascular Accident

## Results

### Demographics and patient characteristics

Our study included 1,572,815 individuals, with 22,230 (1.4%) in the PTX group and 1,550,585 (98.6%) in the non-PTX group (Table 1). The PTX group consisted of more Hispanic patients (31.8% vs. 21.3%) and fewer White patients (44.2% vs. 51.1%) than the non-PTX group (*p* < 0.001). The PTX group also had a higher proportion of males (65.8% vs. 51.6%, *p* < 0.001). Patient ages differed between both groups and sexes. There were more patients 50–69 years old in the PTX group (46.7% vs. 37.0%, *p* < 0.001). Additionally, the average age for females was slightly higher in the PTX group (65.9 years vs. 63.1 years), while it was almost identical for males across both groups.

There was a small difference in patient income between the two groups and no difference in insurance. There were more individuals earning less than $49,999 a year in the PTX group than in the non-PTX group (36.4% vs. 34.1%, *p* = 0.004). However, there were no substantial differences in insurance status between the two groups (*p* = 0.113).

There were differences in hospital division distribution, bed size, and teaching status between the two groups. The West South-Central hospital division saw a significantly higher percentage of PTX cases (18.1% vs. 14.2%, *p* < 0.001). There was also a higher proportion of PTX patients at large hospitals (50.4% vs. 46.6%, *p* < 0.001) and urban, teaching hospitals (76.3% vs. 71.5%, *p* < 0.001).

Comorbidity profiles differed between the two groups. PTX patients were less likely to have coronary artery disease (15.0% vs. 17.9%, *p* < 0.001), myocardial infarction (3.1% vs. 4.2%, *p* < 0.001), hypertension (62.6% vs. 64.9%, *p* = 0.001), smoking history (19.6% vs. 25.6%, *p* < 0.001), chronic kidney disease (10.6% vs. 12.6%, *p* < 0.001), hypothyroidism (10.5% vs. 13.3%, *p* < 0.001), depression (7.6% vs. 11.0%, *p* < 0.001), and dementia (5.8% vs. 12.2%, *p* < 0.001). They were more likely to have Type 2 diabetes (41.6% vs. 40.1%, *p* = 0.032), cancer (5.0% vs. 4.2%, *p* = 0.010), and obesity (28.6% vs. 26.4%, *p* = 0.001). There were no significant differences in drug abuse, chronic pulmonary disease, peripheral vascular disease, autoimmune disease, or AIDS prevalence. All demographics and patient characteristics from the univariate statistical analysis are in Table 1, with the PSM results in Table 3.

### In-hospital morbidity and mortality

In-hospital mortality between PTX and non-PTX COVID-19 patients differed significantly. Of the 1,550,585 non-PTX patients, 12.6% died in the hospital. This was markedly lower than the 65.8% mortality rate among the 22,230 PTX patients. The adjusted odds ratio (AOR) for in-hospital mortality was significantly high at 15.9 (95% CI 14.9–17.0), indicating a substantially higher risk of in-hospital death for PTX patients (Table 2). PSM of 44,460 COVID-19 patients (half with PTX, half without) revealed the in-hospital mortality rate was 14.4% for non-PTX patients but 65.8% for PTX patients.

PTX patients experienced a significantly higher rate of in-hospital complications. For example, the AOR for acute liver failure and sudden cardiac arrest were 7.2 (95% CI 6.4–8.1) and 7.0 (95% CI 6.5–7.6), respectively. Additionally, these patients experienced acute kidney injury (AKI) (AOR 4.4, 95% CI 4.1–4.6), venous thromboembolism (AOR 3.5, 95% CI 3.2–3.8), cardiogenic shock (AOR 4.2, 95% CI 3.5–5.1), hemodialysis (AOR 4.0, 95% CI 3.6–4.3), cerebrovascular accident (AOR 2.9, 95% CI 2.5–3.3), and tracheostomy placement (AOR 14.1, 95% CI 12.8–15.5). PSM yielded similar results. PTX patients experienced a higher prevalence of acute liver failure (AOR 6.7, 95% CI 5.0–9.0), sudden cardiac arrest (AOR 7.4, 95% CI 6.0–9.2), and vasopressor use (AOR 7.5, 95% CI 6.2–9.1). PTX patients had greater incidences of cerebrovascular accidents (AOR 3.0, 95% CI 2.3–4.0) and an increased need for mechanical circulatory support (AOR 11.5, 95% CI 6.4–20.6). They also showed a greater incidence of AKI (AOR 4.1, 95% CI 3.7–4.5), venous thromboembolism (AOR 3.2, 95% CI 2.7–3.8), cardiogenic shock (AOR 5.1, 95% CI 3.3–7.9), and hemodialysis (AOR 3.7, 95% CI 3.2–4.4) (Table 4).

Of note, the need for chest tube placement was remarkably higher among PTX patients at 59.8% compared to 0.6% in non-PTX patients, with an AOR of 244.9 (95% CI 226.3–265.1) (Table 2). Results from PSM showed increased rates of both tracheostomy placement (AOR 14.2, 95% CI 10.5–19.0) and chest tube placement (AOR 231.3, 95% CI 163.1–328.0) (Table 4). PSM showed similar trends for mechanical ventilation, with PTX patients having much higher rates of mechanical ventilation, even when separated into invasive (AOR of 23.1; 95% CI 20.4–26.2) and non-invasive (AOR 3.8; 95% CI 3.3–4.5) support (Table 4).

### In-hospital quality measures

The in-hospital quality measures of total charges for each patient and LOS were compared between the PTX and non-PTX cohorts. PTX patients required considerably more resources as reflected in total charges. The mean total hospitalization charge for PTX patients was notably higher than for non-PTX patients ($435,508 vs. $86,822), with a difference in adjusted total charge for PTX patients being $336,782 greater. The mean LOS was also extended for PTX patients at 23.6 days, compared to 7.8 days for non-PTX patients; the adjusted LOS was 15.3 days longer for PTX patients (Table 2). Data from PSM corroborated this trend: PTX patients had a significantly higher mean total hospitalization charge at $435,508 compared to $96,668 for non-PTX patients, which is an adjusted total charge of $320,978 more. Similarly, the mean LOS for PTX patients was 23.6 days, compared to 8.6 days for non-PTX patients, marking an adjusted LOS 14.9 days longer (Table 4).

### Disposition

Hospital dispositions between the two groups differed significantly. More non-PTX patients left against medical advice (1.1% vs. 0.3%), were discharged to home health care (13.1% vs. 5.4%), were discharged routinely (51.5% vs. 7.6%), or were transferred to another care facility (18.6% vs. 16.4%). In contrast, more PTX patients were transferred to a short-term hospital (3.0% vs. 4.5%). PSM produced similar results in outcomes. For example, 0.9% of non-PTX patients left against medical advice compared to 0.3% of PTX patients. These differences, reflected in both AOR and PSM data, highlight the impact of PTX on the course of recovery in COVID-19 patients (Tables 2 and 4).

## Discussion

We found that the average incidence of PTX in COVID-19 patients was 1.41% out of 1.5 million people affected in 2020, and patients who developed PTX had a significantly higher mortality rate, greater resource utilization, longer LOS, and higher costs of care. This cohort was more likely to be male, obese, and Hispanic, illuminating socioeconomic, racial, and gender disparities. Patients with PTX required more aggressive treatment interventions and had longer hospital stays, highlighting a need for COVID-19 prevention and treatment optimization to minimize the risks of developing PTX.

COVID-19 positive individuals with PTX require more support, have longer hospital stays, have higher costs of care, and use more hospital resources. We found that this cohort was more likely to need mechanical circulatory support and interventions like chest tube placement, and were more likely to experience a long, complicated hospital stay that resulted in tracheostomies. Their mean LOS was nearly 15 days longer than the LOS of non-PTX patients. Previous studies have shown that critically ill patients with COVID-19 often require invasive procedures, including central venous access, that possess an additional risk of iatrogenic PTX [24]. Wang et al. found that PTX was associated with high mortality, poor prognosis, and prolonged LOS [9]. Costs of care are related to both the LOS and amount of support a patient requires; the PTX patients in our study had significantly higher costs of care than non-PTX patients, with an adjusted mean total hospitalization charge $320,977.60 higher.

We also compared mortality between the two cohorts. We found that hospital mortality in COVID-19 patients with PTX admitted in US hospitals between January–December 2020 was nearly 14 times higher than in patients without this complication. Geraci and colleagues reported similar outcomes of a 58% mortality rate in PTX patients and 63% in MVPs [17]. Similarly, in another multicenter study, the odds of mortality in patients with COVID-19 related PTX was 7.15 compared to those who did not have PTX [25].

PTX is closely correlated with a patient needing mechanical ventilation, and both are correlated with high mortality rates. The higher mortality rate in our study is associated with high rates of invasive mechanical ventilation (77% vs. 15%). These results are validated by a large multicenter study of 842 critically ill patients (71% MVPs) that showed that PTX patients had a higher mortality rate (63% vs. 49%, *p* = 0.04), with the odds of in-hospital death increased nearly two-fold [21]. PTX patients often have more severe illness and spend more days on mechanical ventilation, placing them at higher risk of needing additional interventions, like tracheostomy and mechanical circulatory support [17, 26]. Invasive interventions increase the risk of multi-organ failure or organ damage, resulting in longer hospital stays and greater mortality [17]. The extensive support needed by these patients highlights the value of understanding the causes of PTX so it can be prevented.

Understanding the pathophysiology of COVID-19-associated PTX may help us understand why this progression is associated with such high mortality rates. Developing COVID-19-associated PTX can be attributed to pathophysiological changes associated with COVID-19 and disease progression. One of the proposed pathological mechanisms, along with a COVID-19 hyperinflammatory state, include viral tropism to peripheral lung pneumocytes with a predisposition to peripheral bronchoalveolar communication, consequently leading to PTX and pneumomediastinum, along with barotrauma associated with positive-pressure ventilation [27]. Contrary to this, studies have shown that PTX tends to occur later in the disease course when the hyperinflammatory state has decreased, likely from the dysregulation of lung repair [28]. COVID-19 specific therapies potentially reduce viral clearance and interrupt the innate remodeling and repair process that may help prevent PTX [18].

Multiple factors are associated with developing PTX, including chronic pulmonary disease, smoking, previous history of lung disease, and prolonged intubation [20, 28]. Notably, both cohorts in our study had nearly identical rates of pre-existing comorbidities. However, contrary to most studies, there was a higher proportion of obese patients with COVID-19 and PTX. Obese patients are particularly at risk for developing COVID-ARDS and having poor outcomes [29]. Although the association between body mass index (BMI) and PTX has not been established in patients with COVID-19, patients who develop spontaneous PTX tend to have a lower BMI [30]. Similarly, a matched case-control study of 427 patients showed that PTX patients had a lower average BMI (22.4) than non-PTX patients (24.5), which falls under the lower range compared to our population [31]. In contrast, a large study composed of 9800 COVID-19 patients, of which 67 developed PTX, two thirds of the affected patients had a BMI over 25 [10]. Due to the conflicting evidence, additional research is needed to thoroughly assess the association between COVID-19, PTX, and other comorbidities. However, it can be hypothesized that obesity, being a well-established risk factor for severe C-ARDS, may increase the risk of complications, including PTX.

Determining a gender difference in the incidence of PTX associated with COVID-19 is complicated. The risk of primary spontaneous PTX is higher among males between the ages of 25–34 years, with risk increasing with patient height [32]. In our study, males were nearly two times more likely to develop PTX than females. One reason for such differences could be the overall incidence and severity of COVID-19 being higher in the male population than the female population [33]. The Centers for Disease Control and Prevention suggests the psychological, social, and behavioral differences in males may play an important role in overall exposure of SARS-COV-2, comorbidities (e.g., smoking), treatment initiation, compliance, and overall mortality [34]. Smaller retrospective studies present similar findings [33, 35]. Records in NHS’s OpenSAFELY health analytics platform show being male is a risk factor for COVID-19-related deaths [36]. There is preclinical evidence that sex is important in the genetic and hormonal modulation of immune responses. For example, biological sex influences the expression and regulation of angiotensin-converting enzyme 2, the main receptor SARS-CoV-2 uses to enter cells [37]. Ineffective anti-SARS-CoV-2 immune responses, coupled with higher pre-existing comorbidities, could explain the increased mortality risk [38]. Hence, it can be extrapolated that being male is a risk factor for developing severe COVID-19 and associated complications, including PTX. However, more studies are required to further evaluate and risk stratify the gender difference in incidence and outcomes associated with PTX.

The COVID-19 pandemic has highlighted long-standing, broad, and pervasive inequities between racial and ethnic minorities. Although the White population overall was most affected, Hispanic patients who were affected by COVID-19 had higher rates of PTX compared to other ethnicities (32% vs. 21%, *p* < 0.001). Racial and ethnic minorities often face barriers to healthcare, including socioeconomic factors, overcrowded living situations, high pre-existing comorbidities, systemic implicit bias, and vaccine disparities. These barriers can lead to high COVID-19 rates and thus, high ICU admission rates—shedding light on the rates of complicated COVID-19-ARDS in the Hispanic population [39, 40]. Further research is imperative to gain a more comprehensive understanding of the racial distribution of COVID-19. While existing studies have highlighted disparities in infection rates, severity of illness, and mortality among different racial and ethnic groups, there are still many unanswered questions and evolving dynamics, including vaccine equity and intersectionality.

### Limitations

There are several limitations of our study to consider, including inherent limitations due to the retrospective nature of the study. One significant limitation is the inability to determine the exact timing of PTX in relation to the initiation of mechanical ventilation, owing to the constraints of the database used. Furthermore, the NIS database employed for this research is administrative in nature, which may not fully encapsulate the intricate details of a patient’s hospitalization, such as history, physical examination findings, laboratory values, and imaging/pathological investigation reports. Additionally, these inpatient outcomes may not accurately reflect outpatient trends. It is also crucial to emphasize that these results were compiled prior to the development and widespread administration of effective COVID-19 vaccines. As such, they may not accurately depict current hospitalization patterns in the era of COVID-19 vaccination.

## Conclusions

In our comprehensive analysis of 1.5 million COVID-19 patients during the year 2020, a significant association was established between the presence of PTX and a multitude of adverse outcomes. Specifically, COVID-19 patients who developed PTX exhibited elevated rates of in-hospital mortality, increased reliance on mechanical ventilation, greater vasopressor use, heightened occurrence of AKI, venous thromboembolism, and cerebrovascular accidents, as well as elevated procedural intervention rates, causing increased resource utilization. Risk factors for COVID-19 patients developing PTX were being Hispanic, male, or obese. These findings collectively underscore the profound impact of PTX in the context of COVID-19, emphasizing the critical need for early recognition, preventative measures, and optimized management to mitigate the associated risks, reduce treatment costs, and improve patient outcomes.

### Electronic supplementary material

Below is the link to the electronic supplementary material.

Supplementary Material 1

## Data Availability

We queried our data from the 2020 edition of the national inpatient sample, part of the Healthcare Cost and Utilization project. NIS is accessible to the public, interested researchers can retrieve the data directly via the HCUP website https://hcup-us.ahrq.gov/tech_assist/centdist.jsp, accessed on 01 January 2024).

## Abbreviations

AKI: Acute kidney injury
AOR: Adjusted odds ratio
ARDS: Acute respiratory distress syndrome
BMI: Body mass index
CI: Confidence interval
COVID-19: Coronavirus Disease 2019
ICD-10-CM: International classification of diseases 10th—clinical modification
LOS: Length of stay
MVPs: Mechanically ventilated patients
NIS: Nationwide Inpatient Sample
PSM: Propensity score matching analysis
PTX: Pneumothorax
SD: Standard deviation
